# Perceived Health System Barriers to Tuberculosis Control Among Health Workers in South Africa

**DOI:** 10.5334/aogh.2692

**Published:** 2020-02-12

**Authors:** Prince A. Adu, Annalee Yassi, Rodney Ehrlich, Jerry M. Spiegel

**Affiliations:** 1 School of Population and Public Health, University of British Columbia, Vancouver, CA; 2 British Columbia Centre for Disease Control, Vancouver, CA; 3 School of Public Health and Family Medicine, University of Cape Town, South Africa, ZA

## Abstract

**Background:**

The healthcare workforce in high tuberculosis burden countries such as South Africa is at elevated risk of tuberculosis infection and disease with adverse consequences for their well-being and productivity. Despite the availability of international guidelines on protection of health workers from tuberculosis, research globally has focused on proximal deficiencies in practice rather than on health system barriers.

**Objective:**

This study sought to elicit perceptions of informed persons within the health system regarding health system barriers to protecting health workers from tuberculosis.

**Methods:**

Semi-structured interviews were conducted with 18 informants active in spheres related to workplace tuberculosis prevention and management in South Africa. Interviews were audio recorded and transcribed verbatim, validated and analysed to derive emergent themes. Responses were analysed using the World Health Organization building blocks as core elements of a health system bearing on protection of its health workforce.

**Findings:**

The following health system barriers were identified by informants: leadership and governance were “top-down” and fragmented; lack of funding was a major barrier; there were insufficient numbers of staff trained in infection prevention and control and occupational health; occupational health services were not comprehensively available and the ability to sustain protective technologies was questioned. A cross-cutting barrier was lack of priority afforded to workforce occupational health associated with lack of accurate information on cases of TB among health workers.

**Conclusions:**

We conclude that deficiencies in implementation of recommended infection control and tuberculosis management practices are unlikely to be corrected until health system barriers are addressed. More committed leadership from senior health system management and greater funding are needed. The process could be assisted by the development of indicators to characterise such barriers and monitor progress.

## Introduction

Health workers, including care-giving professionals and support personnel and students, are an essential component of healthcare systems. Their short supply where disease burden is greatest has prompted recognition of human health resources as a priority global health challenge [1,2]. In September 2018, the United Nations General Assembly High Level Meeting on Tuberculosis agreed on the need to better protect health workers as part of the global campaign against this leading cause of death from a single agent [3]. However, the health and safety of this essential population, particularly in low-resource countries, have been neglected and have received limited attention in health system priority setting [4].

Tuberculosis (TB) transmission in the healthcare workplace has prompted the development and dissemination of numerous guidelines for strengthening TB infection prevention and control (IPC) in such settings and improving health workers’ access to HIV and TB prevention, treatment, care and support services [5]. Nevertheless, a growing body of literature indicates that inadequate implementation of actions to prevent workplace transmission of TB persists in high burden countries generally and South Africa in particular [6,7,8,9,10].

Health workers worldwide have on average a two to three times greater risk than the general population of being infected with *Mycobacterium tuberculosis* and developing active TB [11,12]. This implies large numbers of health workers at risk of occupational infection, in addition to their risk of community infection. In South Africa, where one of the worst HIV-TB epidemics globally has persisted, an annual rate of 1496 TB cases per 100,000 was estimated among health workers for the 2002–12 decade (compared to annual rates of 700–800/100,000 in the general population and rates of <10/100,000 in high-income countries [13]). High rates of annual TB infection have been recorded in South African health workers and medical students [14,15]. Of further urgent concern is that a considerably elevated rate of multidrug resistant TB has been shown in South African health workers relative to a comparison sample of non-health workers [16].

Protecting the health of health workers is the direct responsibility of the health system that employs them. We believe that we need to move beyond documenting the size of the problem of occupational TB risk and the extent to which preventive actions fall short of accepted guidelines, to explicitly consider what barriers to implementation are at play. Further, we need to look beyond the operational level of infection and prevention control and examine barriers inherent in the health system. The objective of the study was therefore to elicit the perceptions of informed persons of the health system barriers to achieving such protection.

## Materials and Methods

### Conceptual framework

Studies documenting shortcomings in implementation of clinical practice guidelines in healthcare typically draw attention to personal, guideline-related factors [17], with adherence to occupational infection control recommendations tending to empirically focus on “proximal” factors at the workplace level. These typically measure the knowledge, attitudes, practices and experiences associated with applying recommended practices, for example, knowledge of policies, receipt of training, symptom screening of patients, or use of respirators [7,8]. While these measures are essential, to avoid the approach characterized as “prisoners of the proximate” [18], we explored the extent to which upstream or “macro” factors constitute barriers to implementation.

Building on an initial open-ended consideration of barriers identified by key informants (KIs), we then applied the World Health Organization (WHO) health systems building blocks framework [2] to characterize identified barriers. This includes six distinct but complementary components: leadership and governance, health information system, financing, health workforce, service delivery, and access to essential medical products and technologies. A schematic approach to applying these building blocks in this setting is given in Table 1. While the health workforce itself constitutes one building block of an integrated health system serving or protecting a general population, we were interested in how such system elements could be applied to consider how the wellbeing of this important subpopulation is itself being supported. In this way, TB risk is used as a lens for considering how the health of the health workforce is being protected – a responsibility we believe is seldom foregrounded in health systems thinking.

**Table 1:** Application of the WHO health system building blocks.

Building blocks	As applied to providing systems to support the health of	

	General population	Health Workers

Leadership & Governance	… across professions, institutions	… within health system and across disciplines to ensure prevention and control of TB risk
*Existence of policy frameworks & oversight, design, accountability*		
Financing	… for all health system components	… for occupational health & safety and/including infection prevention and control (IPC)
*Adequate funds for needed resources, good and services*		
Health Workforce	… for health professionals & support	… for occupational health and IPC professionals and support staff
*Human resources management, skills & policies*		
Service Delivery	… diagnostic, curative & general health services	… for prevention, measurement and mitigation of TB risk and effects of the disease in health workers
*Encompassing quality, access, safety and coverage of provided services*		
Access to Medicines and Technology	… for drugs, devices and other supplies	… for environmental control, personal protective equipment, and access to diagnosis and effective medicines for health workers
*Ensuring access to needed equipment and materials*		
Information System	… for population health status & service use; trends and associations	… for health worker TB status and workplace risk; trends and associations
*Production, analysis, dissemination and use of timely & reliable information*		

Several overlapping disciplinary approaches contribute to the prevention of TB transmission and protection and management of affected workers, including infection prevention and control, occupational health, and patient-orientated clinical programming such as FAST (Find cases Actively, isolate Safely, Treat effectively) [19]. In this study, interview questions were framed within an occupational health approach that considers the well-being of workers across the spectrum of primary, secondary and tertiary prevention measures. We chose this approach as health facilities have statutory responsibilities under occupational health legislation in South Africa to provide such protection to health workers across this whole range [20].

### Study design

Given the exploratory nature of our research question, we used qualitative research methods. The key informant method is especially appropriate in circumstances where little or no established evidence base or indicators are available to analyse phenomena of interest. Qualitative research methods were used in line with the Consolidated Criteria for Reporting Research (COREQ) checklist [21].

### Setting, participants and interviews

Twenty potential key informants were approached, two of whom did not respond to further communication. Between October and December 2016, semi-structured interviews were conducted with 18 key informants whose current position entailed the care, prevention, control and/or management of TB or were indirectly involved in TB prevention or policy (Table 2). They included ten government employees with responsibility for TB control (designated as “G” in the quotes below), four academic experts (“A”), two TB advocacy group members (“N”), a legislator (“L”), and a hospital leader (“H”). The informants included 11 women and seven men and were based in the provinces of Gauteng, Western Cape and KwaZulu-Natal. All were presumed to have in-depth knowledge of the problem of occupationally acquired TB through their professional careers or (in two cases) personal experience of the disease and subsequent activism. Table 2 provides the key informants’ category of expertise or practice, profession and the mode of interview.

**Table 2:** Location of key informants.

Reference	KI Category	Designation	Mode of Interview

G1	Provincial and national government authorities	Senior level manager in charge of occupational hygiene in a provincial health department	In person
G2		Mid-level manager in charge of occupational hygiene in a provincial health department	In person
G3		Mid-level manager in charge of occupational hygiene in a provincial health department	In person
G4		Mid-level manager in charge of occupational hygiene in a provincial health department	In person
G5		Senior level manager in charge of wellness programme in a provincial health department	In person
G6		Senior manager in charge of TB, national Department of Health	In person
G7		Senior manager in charge of hygiene and health, national Department of Labour	In person
G8		Senior manager, national Department of Public Service	In person
G9		Senior manager, National Institute of Occupational Health	In person
G10		Occupational medicine specialist, National Institute Occupational Health	In person
A1	Academic experts (university based)	Professor Emeritus in occupational medicine	Skype
A2		Professor in occupational health	In person
A3		Professor in public health medicine	In person
A4		Professor in occupational health	In person
N1	Occupational TB advocacy group	Survivor of MDR TB and TB advocate/former health worker	Telephone
N2		Survivor of MDR TB and TB advocate/health worker	Telephone
H1	Hospital heads	Head of a large TB referral hospital	In person
L1	Health legislator/politician	Member of national parliament	Telephone

Participants were recruited via email using two approaches. First, purposive sampling was used to identify 14 potential key informants. Subsequent recruitment was conducted using a snowball sampling technique after a preliminary analysis of the gathered data. Participants were recruited until data saturation was reached.

Fifteen semi-structured interviews were conducted in person at a work location or other location of the participant’s choice; a further two were conducted over the telephone and one via Skype. The interviewer (author PAA) was then a doctoral candidate with a Masters of Public Health as well as a Masters in International Development, and ten years of experience in conducting qualitative research in the Global South.

All participants were informed prior to the interviews of the research goals via recruitment letter, consent form and interview script. Consent was confirmed at the interview and participants were reminded at the interview that participation was voluntary and that they could withdraw at any time. Interviews lasted 35–60 minutes. Participants were briefed that the interest of the research was in “upstream” or “macro factors” affecting occupational health and infection control programmes. These factors were distinguished from those under the direct control of the individual (“micro” level) or facility (“meso”). Questions were mostly broad, but included specific reference to political, economic, macro, organizational and cultural barriers; and to resource allocation, governance, education, and awareness of TB programmes and related policies.

Interviews were audio recorded and transcribed verbatim. After each interview, the interviewer repeated the responses (from the field notes) for the participant to correct or confirm as needed. Two of the participants were re-contacted during data transcription to clarify content.

### Data analysis

The NVivo^®^ Version 11 qualitative data management software was used to organize data collected from the interviews. As a first step, emergent codes with reference to the objectives of the study were identified within each interview and aggregated into new categories with the assistance of two other doctoral candidates, one in communication studies and the other in nursing. Identified themes were then grouped with reference to the WHO building block dimensions described above.

## Results

We identified seven themes: one cross-cutting and five directly corresponding to the WHO health systems building block framework.

### Lack of priority afforded to protecting worker health

Informants largely agreed that occupational health and (to a lesser extent IPC) is not prioritised in health facilities in South Africa. Various observers indicated that knowledge about policies and guidelines is not the problem, but rather that effective leadership championing occupational health was lacking.

*[There is] a shortage of resources and lack of involvement of the CEOs because it [occupational health] is not a priority* (A1).

*We struggle even with managers for buy-in as well as with employees* (G10).

*The senior managers and the political heads may not know or understand occupational health; not that they don’t want to do it, but they are not aware* (A4).

Recounting a meeting with a hospital chief executive officer (CEO), a government official explained this concern in more direct and personal terms.

*One CEO said to me, if we had to choose between an Intensive Care Unit (ICU) nurse and IC (infection control) nurse, the CEO will take an ICU nurse. Because there are not enough resources, they would rather channel resources to serving the public than those people assisting the public* (G7).

Others pointed out that even with IPC, protecting patients is prioritized over occupational health measures to protect staff. There was also failure to link health worker retention with safe working conditions.

*Generally, the [health] organizations do not prioritise employee workplace services. When people talk about IC, they think only of patient infection. So, they look at the patient side. …. Even if they manage their human resources, it may be the issue of resignation and people leaving but they don’t look at why people may be leaving… it may be an OH issue that makes people leave…* (G6).

*To retain your health professionals within health institutions, there are really serious health system changes that are needed in terms of conditions of service, workload, safety, etc.* (L1).

The high TB burden in the South African general population was cited in relation to risk. The lack of accurate information about TB occurring among health workers (and by implication their excess risk) was identified as a specific barrier to systemic awareness and action.

*I am very convinced that more than half of our health care workers acquire their TB from the community. It is good to look at the workplace factors, but you need to look beyond the workplace and think of the primary sources of community-acquired sources of TB* (A2).

*There were two support staff who were diagnosed (with MDR TB) but they contracted it from the community; we found out that the source was not hospital* (H1).

*The true risk (of TB) among health care workers is not known because cases are not properly identified. There is uncertainty and this uncertainty feeds into the organizational response* (A1).

The remaining emergent themes were encompassed by the WHO Building Block framework and are presented in the next section.

### Governance and leadership

Governance has been defined as the processes which “determine(s) who has power, who makes decisions, how other players make their voice heard and how account is rendered” [22]. Inappropriate governance of occupational health was identified as a major factor inhibiting implementation of protective and supportive measures in respect of occupational TB. Some interviewees pointed out that such governance often falls organizationally under the health facility’s human resources management department (HR). This creates problems because HR officials may lack knowledge or clinical understanding of health worker health issues.

*You actually need clinicians running (OH services in health facilities), not the HR department* (A1).

When asked whether health workers themselves had any influence on the governance of TB prevention in the workplace, there were mixed opinions, with the majority of informants contending that involvement of health workers was minimal.

*They (health workers) don’t (get involved). There are Health and Safety Committees, but from what I’ve seen this is an exercise in futility. The attention is on human resource development not on occupational health* (A3).

A health worker advocacy group member provided a perspective on whether health workers can influence policy at the national (rather than at facility or local level).

*There is no direct access to the decision makers around TB implementation policy in SA. There is no open-door policy from the TB directorate at the national level and from previous interactions we’ve had, they have not been particularly receptive to advice from outside* (N2).

On the question of policy development and implementation, an academic expert identified policy blockage at lower levels (i.e. provincial as opposed to national) of the South African health system.

*It’s not like there is no structure in place. …We will get a small committee to draft the policy. Then that draft will be circulated to provinces…. So, they will determine if this is implementable …. Then we modify based on it. Then we have a broader consultation, provinces and other key stakeholders and then finalize it. But we find that when we send this to the provinces they don’t consult. They just sit on the document and when you call to say we are waiting for your inputs then they come back and say you didn’t cross your “t’s” etc. and not look at content* (A4).

Another aspect of policy development and implementation identified was integration of occupational health and IPC.

*To reduce your barriers, you need your policy of OHS (occupational health and safety) and IC (infection control) very close together in place so that they will explain your resources, the networks, the monitoring and also implementation* (H1).

Finally, informants stressed that a “culture of non-compliance” spanned top management as well as frontline workers, harking back to lack of leadership.

*… there is a barrier of non-compliance from high up within the healthcare sector… there is no management authority behind it [implementation]* (G5).

### Financing

The lack of adequate resources was seen to be one of the major barriers to effective implementation of occupational health guidelines or practices or to providing services.

*There is insufficient allocation of resources, budgeting, issues of commitment and accountability accounted for the inadequate OH services* (G4).

*Budget is an enormous factor because you cannot implement proper IC (infection control) at a healthcare institution (without adequate funds)* (G5).

Asked whether OH services were influenced by political forces, a government official believed “there is a political will. The barrier is with resources” (G9).

### Health workforce for occupational health and IPC

The shortage of well-trained staff dedicated to occupational health and IPC was identified as a major barrier. Explaining the limitations of even the best policies in a human resource constrained environment, an informant noted that:

*You can have wonderful policies, but if you don’t have the human resources do it, you are going to be stuck. So, a lot depends on appropriately trained personnel* (L1).

Many interviewees pointed out that IPC and occupational health are largely nurse-driven in SA, the latter particularly given the lack of occupational medical practitioners (physicians) in the public sector.

*When you go to the hospital, you will find an occupational health (nurse) practitioner. The biggest problem is they are not creating posts for occupational medicine specialist or clinicians with at least a diploma in occupational health in the district hospitals, to be able to provide a lot more than what is being provided. So the young ones are not motivated to specialize in this area. Doing things like annual medical surveillance, screening for infectious diseases, doing risk assessment to inform the kind of medical surveillance is not happening because they don’t have the qualified people to do it. They would rather take a specialist in obstetrics because of a big maternal load than a specialist in occupational health specialist* (A3).

Informants stressed the need for capacity building and strengthening in the prevention and control of occupational TB, and qualified personnel in the provision of these services.

*Programmes … need a dedicated person. You need a person designated for OHS* (H1).

*We need enough OH and IC health workers to deliver the needed occupational health services* (G7).

### Service delivery to health workers

Key informants expressed the need for an environment where treatment for tuberculosis in health workers is not just provided free of charge but also given under confidential conditions. A government official expressed concern about health workers’ preference to seek care at sites other than their workplace’s occupational health units due to confidentiality concerns.

*The health care worker won’t want to go to the clinic where he/she works, so would want to go to the private clinic but they don’t treat TB* (G8).

Becoming known as someone with TB not only exposes the employee to stigmatisation as a TB sufferer (e.g. potentially contagious, association with HIV), but may also be seen by the health worker as threatening their job security. It was reported that even in health facilities with a well-functioning occupational health unit, these services may be underutilized for TB as employees seek treatment outside their system in which they work.

*There is still fear of reporting because of fear of losing your job, or people gossiping about you* (A1).

*I got TB from work exposure and no one knew about it because I was ashamed* (G2).

*There is no confidentiality in the hospitals* (G3).

An important reason for the under-reporting of their TB by health workers is fear of lack of confidentiality, for example, that their personal health records could be accessed by their colleagues.

*Confidentiality is [indeed] a big barrier* (G10).

### Protective technologies

The precarious physical environment in which health workers work was pointed out by many informants as an enabler of nosocomial TB transmission. A former health worker who had suffered occupational MDR TB lamented the poor environmental controls and the fact that personal protective equipment was either unavailable or not worn by health workers in this high-risk environment.

*We didn’t have windows. No proper ventilation. … (We) weren’t using N95s. Infection control wasn’t good at all* (N1).

A group whose risk of occupational TB does not receive much attention is non-clinical support staff.

*We need to pay attention to the porters, the administration clerk, the cleaners, the ward clerks. They are the first contacts. That’s where you need proper ventilation* (G1).

The problems in the use of IPC technology were also highlighted. One informant questioned the evidence for the effectiveness of upper room germicidal ultraviolet light systems, noting that they could create a false sense of protection among health workers. Installation and maintenance of such technology were identified as the problem by others.

*Sometimes it’s not so much of the technology but how it is applied. They (UV lights) were wrongly administered from an engineering point of view. Others at the wrong angle, not maintained, others the bulb not at the right UV emissions, wavelength* (G8).

## Discussion

Early international guidelines, mainly from the US Centers for Disease Control (CDC) and WHO, used by countries such as South Africa to develop national and local guidelines, tended to focus on specific technical practices with presumed universal applicability. Recognition of upstream or system factors which might hinder or support implementation found expression only in later documents such as the WHO 2009 guidelines which added “national and sub-national level managerial activities” to the facility level. These covered overarching activities such as a comprehensive budgeted plan, health facility design, surveillance, advocacy, monitoring and research and were carried over into the 2019 guidelines [23] in somewhat different language highlighting the importance of system enablers. The purpose of this study was to give this general notion more practical content by specifically examining how system factors are perceived by key actors in a high TB burden health system.

Cutting across the building blocks framework, participants identified lack of priority afforded to occupational health and underestimation of risk to the health workforce as systemic barriers. These resist easy measurement and their identification requires both a critical reading of strategy and policy and an understanding of what is happening at the political and operational level. Hesitancy in investing resources in primary occupational prevention of TB may stem, at least in part, from uncertainty about occupational attribution in individual cases [20].

Informants identified underreporting as an important reason for the lack of awareness of managers (and of health workers) of the extent of increased TB risk in the workplace. While there is a general statutory requirement for medical practitioners and employers to record and report occupational disease under labour legislation, in practice compliance is poor [20]. TB is a notifiable disease under health legislation, but occupational TB is not routinely recorded. The number and location of health workers who suffer from TB annually in South Africa is therefore unknown, a serious barrier to any rational system of control.

Of the WHO building blocks, governance/leadership and financing are the most upstream and set the conditions for the other components. Lack of funding was widely identified in our study as a major barrier. While South Africa spends 8.6% of its gross domestic product on health, higher than many middle-income countries [24], the public health sector is under severe pressure in dealing with the HIV/TB co-epidemic and treating 84% of the population with 55% of the total health expenditure, the rest being spent in the private sector. Although the latest South African national strategic plan for HIV and TB includes recognition of health workers as a “key population for TB” [25], it specifies no targeted actions nor funding to address this crisis. Despite South Africa being a recipient of substantial funding from international agencies, specific funding for programmes aimed at reducing *health worker* TB risk is sparse [26]. Moreover, while specific national fiscal and global economic policies were not discussed explicitly with the interviewees, the influence of pressures that limit public sector funding in recipient countries cannot be underestimated [27].

In regard to governance, fragmentation in TB prevention and control activities was widely identified by our informants as a barrier. Although the theoretical value of integrating IPC and occupational health was recognized, our interviewees noted how in practice these functions are part of different operational management and staffing components of the health service. For example, informants pointed out that while IPC was seen by senior management as part of patient care, occupational health was assigned to the human resources function in a number of provincial departments of health. Various studies have shown the value of coordinating practice for the benefit of worker and patient health, for example, in addressing threats such as severe adult respiratory syndrome (SARS) in a high-income country setting [28]. Such coordination is likely to be at least equally beneficial in high-risk low-resource circumstances [19].

Interviewees noted that participation of health workers on TB prevention issues was sparse, despite the nominal requirement of co-management in health and safety legislation. While health worker groupings may participate in consultation activities, there is little evidence of their role, including that of health and safety committees at facility level, in monitoring implementation. Outside agencies are more active in this regard, such as the Treatment Action Campaign [28] and TB Proof, an organisation of health workers, many of whom have survived TB; these groups have been effectively vocal with colleagues from around the world in pressing for a more active worker role in governance [29,30].

The combination of health workforce protection and service delivery is central to the WHO/ILO guidelines recommending priority and free treatment of health workers [31]. Our interviewees agreed that preventing and addressing the consequences of TB transmission among health workers need an adequately trained health workforce dedicated to OHS and IPC. Faced with inability to employ the necessary qualified staff, and particularly occupational medical practitioners, the temptation is for managers to require current staff to take on additional responsibilities for which they are not necessarily trained. Lack of recognition of OHS and IPC as essential health system components in turn exacerbates skills shortage by making these unattractive professional fields in which to obtain qualifications.

Experience of stigma, linked to fear of breach of confidentiality and privacy, are frequent themes in studies of health workers’ attitude to TB risk and reporting, and reflect lack of perceived trustworthiness of the system [29,30]. The reality that staff clinical information may be accessible to co-workers in many settings requires action at the data security level. Stigma includes the experience or belief on the part of the affected health workers that co-workers are gossiping about them, fear being infected by them, or believe them to be HIV positive, accompanied by feelings of shame and even guilt [20,29,30]. Reduction of stigma requires interventions at community, policy and organisational levels in addition to education of health workers that specifically addresses attitudes and fears [32,33].

With regard to protective technology, evidence supports the effectiveness against TB transmission in facilities of properly installed and maintained upper room ultraviolet air disinfection [34]. However, participants expressed skepticism about the ability of struggling public health facilities to achieve the high standards [35] required for sustained effectiveness. This places the focus of concern on the ability of the public health system to manage technology rather than on the technology itself.

Harris et al. [36] have argued that IPC (and by implication occupational health) provides a bridge between disease specific programmes and health system strengthening in high HIV/TB low-resource settings. In this regard, the role of primary prevention in settings with high burdens of infectious disease is evident – in rapid identification and treatment of infectious patients, provision of adequately ventilated and disinfected spaces, and protection of other patients. Less visible impacts of protection of health workers from TB need to be added to these – such as health worker retention [30]. Although health worker shortage is generally acknowledged as a major problem in the South African healthcare system [37], protecting the health of these workers as a retention strategy has not received the necessary attention, even in reviews charged with addressing strategies for overcoming human resource shortages [38].

## Conclusion

If evidence-based technical guidelines are to be applied to protect health workers from TB, the full range of influences on their implementation need to be carefully examined, especially in resource-limited settings, where effects are often the most severe. Importantly, a greater sense of urgency from national leadership is needed, which would include appropriate funding. A national occupational health policy on TB in health workers funded by the WHO and drafted in 2016 has yet to be accepted by the national Department of Health; in this regard, examination of the influence of austerity-promoting upstream political and economic factors on such priority-setting challenges is well warranted. While the informants participating in this study had a range of relevant backgrounds in clinical, research, health service management and/or policy domains, further research should include, inter alia, infection control practitioners, human resource managers and formal labour representatives, as well as practitioners in private sector facilities. The input of individuals with greater knowledge on the impact of influences on national fiscal and global economic policies would also be useful.

A concrete step to promote consideration of the issues we have discussed could be the development of indicators for tracking progress, including budgetary allocations to IPC control measures and number and qualifications of staff trained in occupational health and IPC disciplines. These could supplement (with some overlap) the factors currently monitored in relation to the protection of health workers from infectious disease transmission, i.e. “proximal” administrative, environmental and respiratory protection practices. Future global and national policies and guidelines should include the means for assessing systems factors hindering or promoting effective implementation – with attention to “implementation science” itself meriting particular attention in how global health challenges can best be addressed.
